# Carbon dot incorporated multi-walled carbon nanotube coated filters for bacterial removal and inactivation

**DOI:** 10.1039/c8ra00333e

**Published:** 2018-02-22

**Authors:** Xiuli Dong, Mohamad Al Awak, Ping Wang, Ya-Ping Sun, Liju Yang

**Affiliations:** Biomanufacturing Research Institute and Technology Enterprise (BRITE), Department of Pharmaceutical Sciences, North Carolina Central University Durham NC 27707 USA lyang@nccu.edu +1-919-530-6704; Department of Chemistry, Laboratory for Emerging Materials and Technology, Clemson University Clemson SC 29634 USA syaping@clemson.edu +1-864-656-5026

## Abstract

Multi-walled carbon nanotube (MWCNT) filters incorporated with carbon quantum dots (CDots) or single-walled carbon nanotubes (SWCNTs) were produced for bacteria removal from aqueous solutions and also for inactivating the captured bacteria. TMTP Millipore membranes were used as the base of these filters. The results showed that filters with higher MWCNT loading had higher bacterial removal efficiencies. Filters with a MWCNT loading of 4.5 mg were highly effective at removing bacteria from aqueous solution, resulting in a log reduction of 6.41, 6.41, and 5.41 of *E. coli* cell numbers in filtrates compared to MWCNT filters without coating, MWCNTs filters with 0.15 mg CDot coating, and MWCNTs filters with 0.15 mg SWCNT coating, respectively. Ionic strength played an important role in bacteria removal. A higher NaCl concentration resulted in higher bacteria removal efficiencies of the filters. Both CDot coatings and SWCNT coatings did not significantly affect the MWCNT filter effects (*P* > 0.05). The coatings, especially CDot coatings, significantly inhibited the activities of bacteria retained on the filter surfaces (*P* < 0.05). The inhibitory rates were 94.21% or 73.17% on the MWCNT filter surfaces coated with 0.2 mg CDots or SWCNTs, respectively. These results demonstrated that MWCNT filters with CDot coatings were highly effective to remove bacteria from water and to inhibit the activities of the captured bacteria on filter surfaces.

## Abstract 2

*E. coli* cells captured and inactivated on a MWCNTs–CDots modified filter.

## Graphical abstract

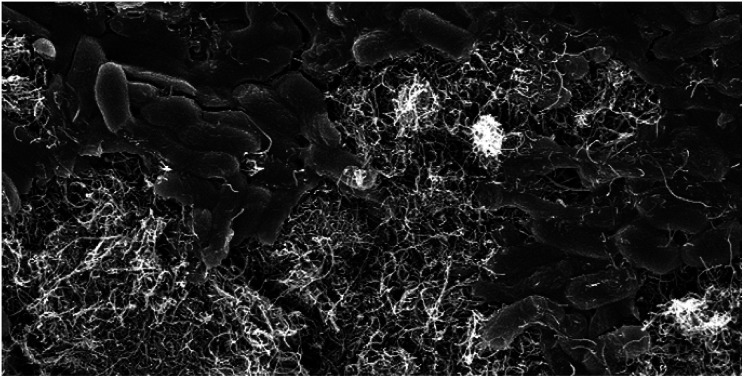

## Introduction

Waterborne diseases caused by pathogenic microorganisms in contaminated water systems lead to 3.4 million human deaths each year.^1^ The World Health Organization (WHO) stated that safe water supplies, hygienic sanitation, and good water management are fundamental to global health. To protect public health, the U. S. Environmental Protection Agency (EPA) has National Primary Drinking Water Regulations which set standards on the maximum contaminant level (MCL) of these pathogens. For example, as *Escherichia coli* O157:H7 is the most problematic pathogen,^2,3^ the EPA regulations require routine sampling of drinking water for testing total coliforms and *E. coli*, and the MCL level is zero for total coliforms.^1^

Various methods and technologies have been investigated to eliminate, inactivate, or directly remove pathogens from water. One of the most commonly used methods is to add antimicrobial chemicals into water. These chemicals include gaseous chlorine (Cl_2_), liquid sodium hypochlorite, chloramine, chlorine dioxide, chloramines, hydrogen peroxide, bromine, *etc.* Although these chemicals have shown high efficacies to inactivate microorganisms, they pose risks in environmental safety and public health,^4^ causing cancers and adverse reproductive disorders by some of these chemicals and their by-products. In other cases, direct contacts with high concentrations of antimicrobial chemical reagents cause extensive damage to human tissues.^5^ Because of the risks posed by these chemicals, the EPA initiated a rule in 1992 to evaluate the need for additional controls for microbial pathogens and disinfectants, with the goal to develop an approach that would reduce the level of exposure to disinfectants, but still keep the high efficacy for controlling microbial pathogens.^6,7^ However, in many cases, low concentrations of the chemical reagents for lower health risks are insufficient for the required antimicrobial effect.^8^

The use of filters without the need to add any chemicals to water is a considerably safer way to remove microorganisms in contaminated water. The traditional membrane microfiltration is effective in the removal of suspended solids, but its usefulness to bacteria removal is limited, showing only about 61% in the bacteria removal rate.^9^ Recent studies have demonstrated that nanomaterials-modified filters could significantly increase the efficiency in microorganism removal.^4^ Specifically, carbon nanotubes (CNTs) have been explored for water purification and desalination purposes to take advantage of the features such as fast water transport, large surface area, ease of functionalization,^10^ and high adsorption capacity. Our previous study and those by others have demonstrated that filters modified by multi-walled CNTs (MWCNTs) could effectively remove bacteria from water samples.^4,11^ On the other hand, single-walled CNTs (SWCNTs) are known to exhibit antimicrobial activities against bacteria and viruses, and are much more efficient than MWCNTs.^12,13^

In order to not only capture or remove bacteria from water, but also inactivate the captured bacteria, the development of effective filters with antimicrobial function is highly valuable. In this regard, the incorporation of antimicrobial agents with CNTs in the modification of filters should be a feasible strategy. Filters coated with MWCNTs and SWCNTs or incorporation of other agents were reported to show some antimicrobial activities.^4,14^

Carbon dots (CDots), another class of carbon-based nanomaterials, have recently been discovered as potent antimicrobial agents with visible-light activation. CDots are small carbon nanoparticles with surface passivation, each having a core–shell structure of a carbon nanoparticle core and a thin shell of soft materials (organic or biological species), with a size profile of less than 10 nm in diameter.^15,16^ There is now substantial experimental evidence suggesting that CDots are similar to conventional nanoscale semiconductors in photoexcited state properties and associated redox processes, which afford CDots to exhibit photocatalytic activities, with diverse catalytic reactions induced by photogenerated electrons (and corresponding holes) on the dot surface.^17^ Our group has reported that the photoinduced redox processes in CDots are responsible for their strong photo-activated antibacterial activities,^18^ and that the antibacterial effectiveness is correlated with fluorescence quantum yields of CDots.^19^ It has also been demonstrated that the optical and redox properties of CDots can be altered or improved substantially by various surface passivation and/or functionalization schemes.^20–23^

The goal of this study was to combine the excellent adsorption characteristics of MWCNTs with the antimicrobial activities of CDots for filters with the dual function of capturing or removing bacteria from water and inactivating the captured bacteria on the filters. Commercially acquired Millipore membranes were used as the base filters. MWCNTs were used to coat the membranes, and the coated filters were grafted with antimicrobial CDots. For comparison, filters co-coated with both MWCNTs and SWCNTs (for additional antimicrobial activity) were also evaluated. The loading amount of MWCNTs, SWCNTs, and CDots, and the effects of filter's operation conditions were tested in terms of the bacteria removal and inactivation efficiencies.

## Materials and methods

### Preparation of MWCNT filters, MWCNT–SWCNT filters, and MWCNT–CDots filters

MWCNTs were purchased from NanoIntegris Inc. (Skokie, IL, USA) and used as received without further purification (purity > 95 wt%). According to the description from the manufacturer, the tube outer diameter, inner diameter, and length were 10–20 nm, 3–5 nm, and 10–30 μm, respectively. The specific surface area was 233 m^2^ g^−1^.

The preparation procedures of MWCNTs coating on filters were similar to those described in our previous publication.^4^ Briefly, isopore polycarbonate hydrophilic membrane filters (TMTP membranes) were purchased from EMD Millipore (Billerica, MA) with a diameter of 25 mm and a pore size of 5 μm. MWCNTs solutions at the concentration of 3 mg mL^−1^ were prepared by suspending MWCNTs in 50% dimethylsulfoxide (DMSO), followed by sonication for 10 min. The MWCNTs filters were produced by depositing MWCNTs solutions onto TMTP membranes with desired loadings. The filters were air dried for 4 h. DMSO residues on the filters were removed by filtering 2.5 mL of 100% ethanol, and then the ethanol residues were removed by filtering 5 mL deionized water (DI-H_2_O). The flow rates on both filtering steps were controlled at 0.5 mL min^−1^ using a syringe pump.

To prepare SWCNTs-coated MWCNTs filters, SWCNTs solutions (1 mg mL^−1^) with functional group –OH on SWCNT surfaces were purchased from Nanolab. Inc. (Newton, MA). The SWCNT lengths were 1–5 μm. The manufacturer synthesized SWCNTs using a chemical vapor deposition process with high yield and purity, containing little or no amorphous carbon. These SWCNTs contained 95.93% weight percentage of carbon and 4.07% of other elements (Na, Al, Si, S, and Fe). SWCNT–MWCNT filters were produced by depositing the SWCNTs solution with desired volumes onto the pre-made MWCNTs filters. The MWCNT–SWCNT filters were air dried for 1 h and then rinsed with 2.5 mL DI-H_2_O by syringe filtering to remove unattached SWCNTs.

To prepare CDots-coated MWCNTs filters, CDots with 2,2′-(ethylenedioxy)bis(ethylamine) (EDA) as the surface functional molecule were synthesized using the same procedure described in our previous publication.^18^ Briefly, carbon nanopowder sample (1 g) was refluxed in an aqueous nitric acid solution (5 M, 90 mL) for 48 h. The reaction mixture was cooled to room temperature, followed by dialyzing against water for 3 days. The desired carbon nanoparticles were obtained by removing the water from the post-dialysis mixture through centrifugation at 1000*g*. To synthesize the EDA–CDots, the carbon nanoparticles were refluxed in neat thionyl chloride for 12 h, and then the excess thionyl chloride was removed *via* purging with nitrogen. The post-treatment carbon nanoparticles (50 mg) were mixed carefully with dried EDA (500 mg) in a flask, heated to 120 °C, and stirred vigorously under nitrogen for 3 days. The reaction mixture was cooled to room temperature, dispersed in water, and then centrifuged at 20 000 × *g* to collect the supernatant as the EDA–CDots in an aqueous solution. Free EDA and other impurities were removed from the solution by dialysis in membrane tubing (cutoff molecular weight ∼ 500) against fresh water. Detailed procedures and characterization of EDA–CDots were previously reported, size-wisely, EDA–CDots were 4–5 nm in average diameter.^24,25^ Similar to the SWCNT–MWCNT filter preparation, the MWCNT–CDots filters were produced by depositing with desired volumes of EDA–CDots solutions at desired concentration onto the pre-made MWCNTs filters. The MWCNT–CDots filters were air dried for 1 h and then rinsed with 2.5 mL DI-H_2_O by syringe filtering to remove unattached CDots.

### Bacteria cultures and filtration

*E. coli* cells or *B. subtilis* cells were freshly grown in nutrient broth at 37 °C overnight. The cells were harvested by centrifugation, washed twice with 0.85% NaCl solution, and then resuspended in 0.85% NaCl, except those stated specifically in the results section, for further experimental uses.

To evaluate the bacteria capture efficiencies of the filters, 2 mL of cell suspension were filtered through the freshly prepared filters at the velocity of 0.5 mL min^−1^ using a syringe pump. The filtrates were collected and the bacterium numbers in filtrates were determined using the traditional surface plating method on Luria–Bertani (LB) agar plates. The bacterium numbers captured by the filters were calculated by using the cell numbers before filtration subtracting the cell numbers in filtrates after filtration.

To determine the inactivation effect of the coated filters on the captured bacteria on the filter surfaces, each filter membrane was sit at room temperature for 30 min and then immersed in 2 mL PBS buffer in a centrifuge tube, followed by 5 s sonication at an ultrasonic water bath and vigorously vortexing until MWCNTs were detached from the membrane. The obtained suspensions were used to determine the viable cell numbers captured on the filter surfaces by the surface plating method. The inactivation efficiency to the captured cells were calculated as the following equation:
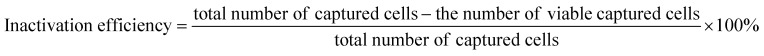


### Imaging of captured cells on filter surfaces

Scanning electron microscopy (SEM) imaging was used to examining the morphologies of *E. coli* cells retained on MWCNTs filters, MWCNTs–CDots filters, and MWCNTs–SWCNTs filters. All the tested filters had 3 mg MWCNTs loadings with or without 0.15 mg CDots or SWCNTs coating on the surface. The captured cells on each filter were first sat at room temperature for 1 h, then fixed overnight by immersing into 1 mL of 4% formaldehyde and 2% glutaraldehyde solution in a 1.5 mL centrifuge tube at 4 °C. The fixative was removed and the filters were gently rinsed with 1 mL DI-H_2_O. The filters were then air dried and coated with gold using Denton Vacuum Desk IV (Czech Republic). The FEI XL30 microscope (Netherlands) was used to take SEM images at the Shared Materials and Instrumentation Facility (SMIF) in Duke University.

### Statistical analyses

Statistical analyses were performed to compare the effects of the filters by the use of the general linear model (GLM) procedure of the SAS System 9.2 (SAS Institute Inc., Cary, NC, USA), with *P* < 0.05 being considered as significant different.

## Results and discussion

### Bacteria removal efficacies of MWCNTs and MWCNTs–CDots coated filters in different buffers

**Fig. 1:**
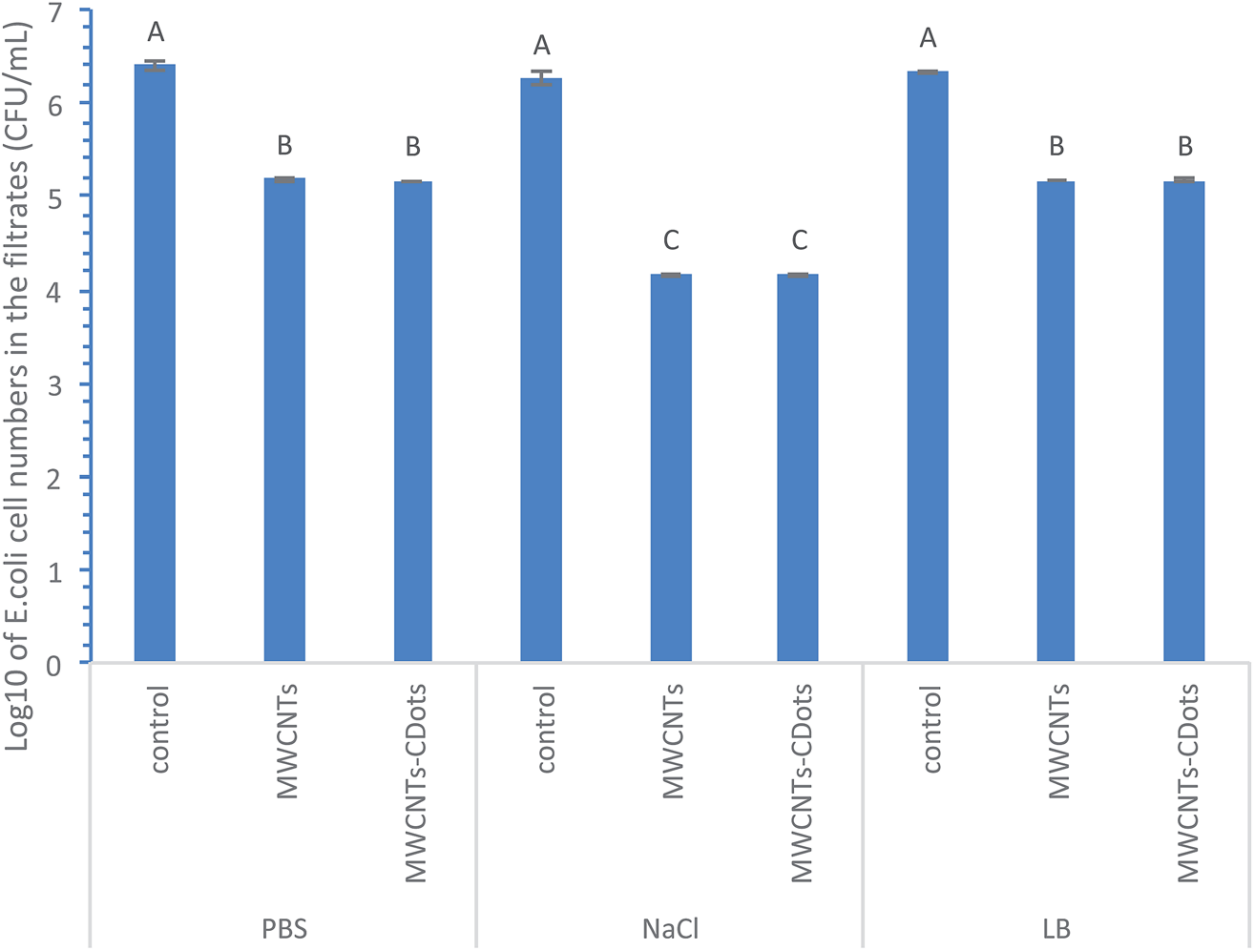
log reduction of *E. coli* cells removed by MWCNTs, MWCNTs–CDots, and SWCNTs–MWCNTs filters in different buffers.

MWCNTs coated and MWCNTs–CDots coated filters were used to test the bacterial removal efficiency to *E. coli* cells in different buffers. Both types of the filters had 3 mg MWCNTs loadings, while the later had 0.15 mg CDots coating on the surfaces. The uncoated TMTP membranes without MWCNTs and CDots were used as the controls. *E. coli* cells were suspended in PBS, 0.85% NaCl, or LB broth. After the filtering process, the viable cell numbers in the filtrates were determined by the surface plating method. Fig. 1 shows the logarithmic value of *E. coli* cell numbers in the filtrates after the filtration using respective filters. The controls were the uncoated TMTP membranes with 5 μm pore size which was much larger than *E. coli* cell size, so all the cells should pass through the TMTP membranes. As such, the controls showed no cell number decrease from the original cell suspensions (∼2.5 × 10^6^ CFU mL^−1^) before filtration. The use of TMTP membranes as the base filters for MWCNTs coating allowed high flow fluxes at low operating pressures while providing a sturdy support for the MWCNTs coating layer.

As shown in Fig. 1, in PBS, the filtrations using MWCNTs and MWCNTs–CDots coated filters both resulted in 1.24 log reduction in the cell numbers compared to the controls. In LB broth, the same log reductions (1.23 log) were achieved by both types of filters for filtering *E. coli* cells, whereas in 0.85% NaCl, the filtrations by both types of filters reached 2.24 log, approximately 1 log more than those achieved in the other two buffers. It is noted that in each buffer, there was no significant difference in the cell number reduction between using the MWCNTs coated filters and the MWCNTs–CDots coated filters (*P* > 0.05).

These results indicated that the buffer played a role in bacteria attachment on MWCNTs' and MWCNTs–CDots' surfaces on the coated filters. It is known that the nature of bacterial attachment to a surface might be determined by the properties of the bacterial cell and the surface properties of the material, as well as the surrounding liquid phase and its influence on the substratum.^26^ Material surface properties that affect bacteria attachment include hydrophobicity/hydrophilicity, roughness, charge, functional group, *etc.*^27,28^ Surfaces conditioned by the migration and adsorption of organic and inorganic molecules, also known as conditioning film, could change their physicochemical properties such as hydrophobicity and surface charge. These property changes could be influenced by the bulk liquid phase surrounding the material and affecting bacterial adhesion.^27,29^ CNTs are highly effective for bacteria adsorption. Solution composition could affect the aggregation state of CNTs as well as bacterial retention in porous media.^30,31^ It was reported that diffusion kinetics of bacterial cells in CNT solution was dependent on the concentration and average diameter of the CNT aggregates and also on the type of CNTs.^32^ In the case of MWCNTs coating on the filters, it was possible different conditioning films with different components were formed and changed the surface properties of the MWCNTs when filtering cells in different buffers, leading to the different extend of bacterial attachment on the MWCNTs or MWCNT–CDots coatings, thus resulting different bacterial removal efficacies when filtering cells in different buffers.

**Fig. 2:**
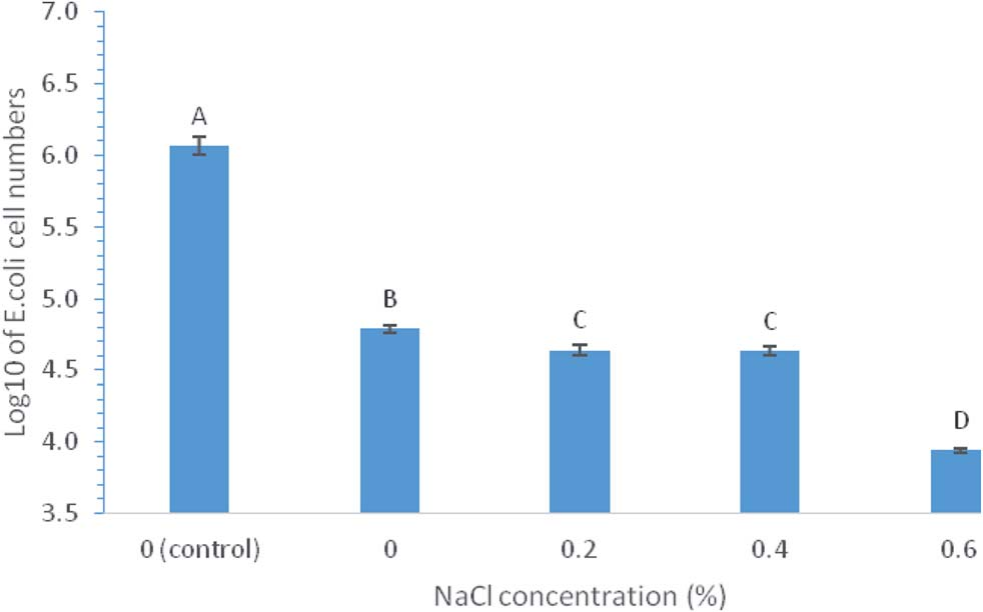
log values of *E. coli* cell number in filtrates after the filtration by MWCNTs filters with 3 mg MWCNTs loading performed in tape water containing 0 to 0.6% NaCl.

Another possible factor that could contribute to the buffer effect on filtration efficiency was the ionic strength in different buffers. To test the ionic strength effects on bacterial removal efficiency by MWCNTs coated filters, *E. coli* cell resuspension in tap water with the addition of 0, 0.2, 0.4, or 0.6% NaCl were tested. The cells in these solutions were filtered through MWCNTs coated filters (3 mg MWCNTs loading). The filtration of the cell suspensions without NaCl resulted in the cell number reduction from 6.11 log to 4.79 log, indicating that MWCNTs coated filters could remove 1.32 log of *E. coli* cells, approximately removing 95.25% of *E. coli* cells (Fig. 2). Cell numbers in all the filtrates were also compared among all the filtrations of cell suspension in water with different NaCl concentrations, the log reductions of *E. coli* cell numbers in filtrates were significantly increased (*P* < 0.05) in the cell suspensions with increasing concentrations of NaCl, as the log reductions of cell number were 1.47, 1.48, and 2.17 in cell suspensions with addition of 0.2, 0.4, and 0.6% NaCl, respectively. It suggested that the increased ionic strength in cell suspensions due to the increasing NaCl concentration improved the filtration efficiency.

This observation demonstrated that MWCNTs coated filters captured more bacteria cells at higher iconic strength, partially explaining the results above on the filtration efficiency in different buffers. Based on literature, bacterial adhesion to surfaces is often explained by the principles of the DLVO theory in dilute NaCl solutions (0.0 to 1.17%), by which the interaction between the cells and the surface is described in terms of attractive van der Waals forces and electrostatic interactions.^33^ Since CNTs are neutral or slightly negatively charged and *E. coli* cells are slightly negatively charged,^34,35^ it was possible that higher ionic strength improved electrostatic attraction force between MWCNTs surfaces and the bacterial surfaces, leading to more cell captures on MWCNTs. Besides, the ionic strength of the surrounding medium might have a direct effect on the cells, thus modifying their rigidity, which in turn influenced their damping effects, and changing the cell surface molecules that mediated the attachment. Similar observations were reported by other studies regarding bacteria–surface interactions, which showed that stronger ionic strength in the solution resulted in greater bacteria adhesion on metal surfaces,^28^ quartz crystal surfaces,^33^ and CNTs surfaces.^36^ Yang *et al.*^36^ studied the influence of CNTs on the transport and retention behavior of *E. coli* cells in packed porous media at both low and high ionic strength in NaCl and CaCl_2_ solutions. Their results demonstrated that CNTs increased cell retentions at high ionic strength (25 mM NaCl and 1.2 mM CaCl_2_), whereas CNTs at low ionic strength (5 mM NaCl and 0.3 mM CaCl_2_) did not affect the retention and transport of the cells.^36^ Brady-Estevez *et al.* reported that MWCNTs filters produced by depositing MWCNTs on 5 μm pore size PTFE membranes could remove more MS2 bacteriophages by increasing ionic strength, from 5.06 log removal at 1 mM NaCl to greater than 6.56 log removal at 100 mM NaCl.^37^ Our results in this study are consistent with these results reported in literature.

### Effects of MWCNTs loading, CDots and SWCNTs loading on bacterial removal efficiency of the coated filters

**Fig. 3:**
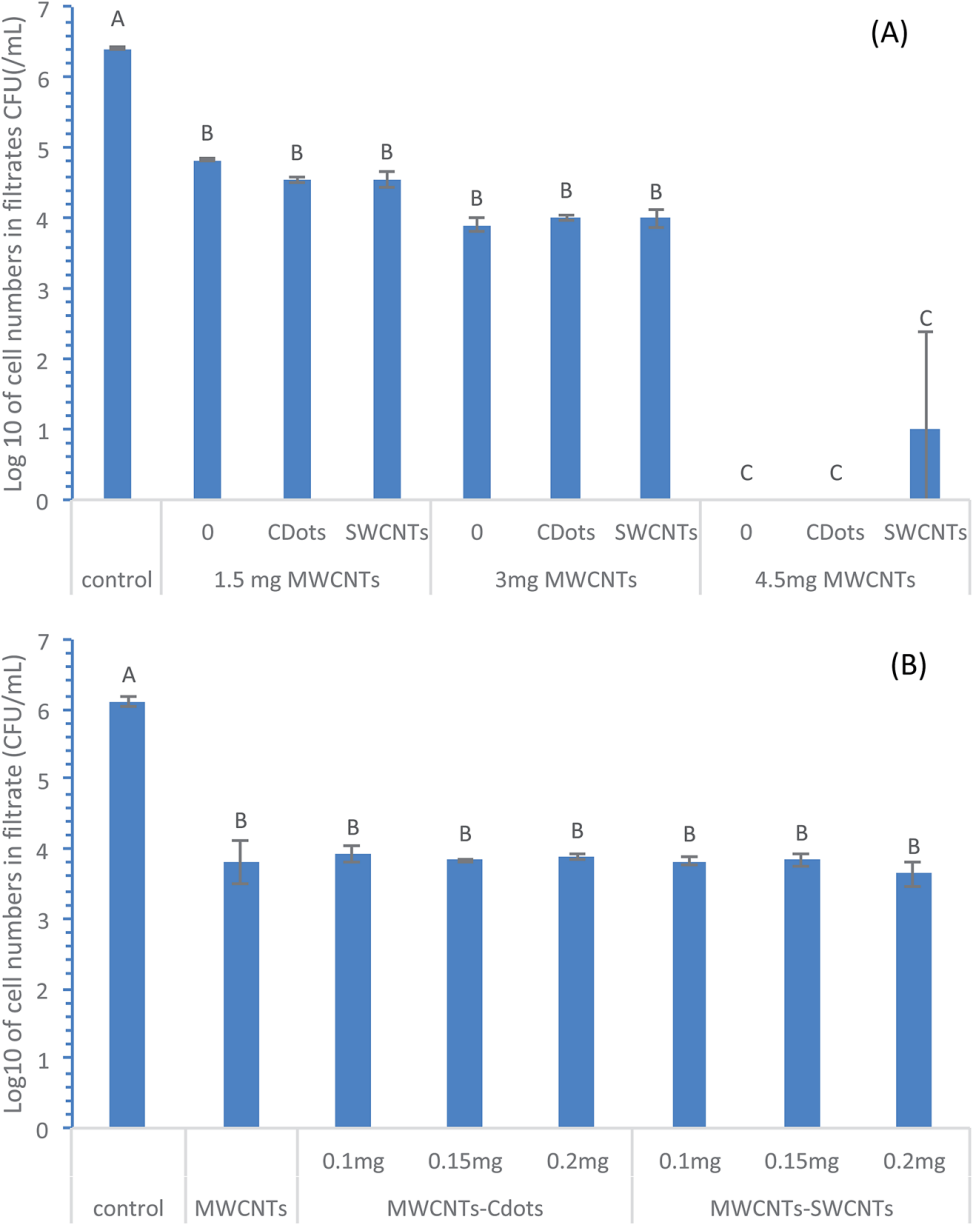
(A) log values of *E. coli* cell numbers in filtrates after filtering through MWCNTs coated filters with three different levels of MWCNTs loading. The MWCNT loadings were 1.5, 3, or 4.5 mg MWCNTs. The surface coatings on the filters were 0.15 mg CDots or SWCNTs. (B) The effects of CDots and SWCNTs loading on the bacterial removal efficiency showing by log reductions in *E. coli* cell numbers after filtration using the coated filters loading with three different levels of CDots and SWCNTs (0.1 mg, 0.15 mg, and 0.2 mg).

To test the effect of MWCNTs loadings on bacteria removal efficacies of different filters, MWCNTs coated filters, MWCNTs–CDots coated filters, and MWCNTs–SWCNTs coated filters, each with three different MWCNTs loadings of 1.5 mg, 3.0 mg, and 4.5 mg were tested. The coating amount of CDots or SWCNTs on MWCNTs–CDots filters or MWCNT–SWCNTs filters was 0.15 mg. Fig. 3A shows the log reductions in cell numbers after the filtrations using the three types of coated filters with different MWCNTs loadings. As shown in Fig. 3, at each level of MWCNTs loading, all three types of coated filters showed significant bacterial removal compared to the controls, with the filters of higher MWCNTs loadings showing higher bacterial removal efficiency, despite there being no significant difference between the filters with MWCNTs loading at 1.5 mg and 3.0 mg (*P* > 0.05). The filters with the highest (4.5 mg) MWCNTs loadings were the most effective and significantly higher than those with lower MWCNTs loadings (*P* < 0.05) for *E. coli* cell removal, even reaching a complete removal by MWCNTs coated filters and MWCNTs–CDots coated filters. The average log reductions in cell numbers achieved by MWCNTs filters, MWCNTs–CDots filters, and MWCNTs–SWCNTs filters with 4.5 mg MWCNTs loading were 6.41, 6.41, and 5.41, respectively. These filters showed high bacterial removal capacities due to the high MWCNTs loading. The results are consistent with previous studies by achieving the similar level of bacterial removal. For example, MWCNTs/Trix buckypapers prepared on 5 μm pore sized PTFE membranes could remove >99% of *E. coli* cells in 0.9% NaCl solution.^38^ Vecitis *et al.*^39^ demonstrated that an anodic MWCNTs microfilter was effective for a complete removal of bacteria. Wang *et al.*^40^ produced filters with the coating of co-poly(propionylethyleneimine)-*co*-ethyleneimine (PPEI–EI) functionalized MWCNTs, and were able to capture higher than 4 log (up to 6 log) of bacterial cells.^40^ Sharing the same characteristics of the outer walls with MWCNTs, SWCNTs filters were also observed to be able to retain *E. coli* cells completely on the filter surfaces.^14^ However, at each level of MWCNTs loading, there was no difference in bacterial removal efficiency when the MWCNTs filters coated with or without CDots or SWCNTs, indicating that additional coating of CDots or SWCNTs did not contribute to the bacterial removal efficiency.

To further confirm the effects of CDots coating or SWCNTs coating on bacteria removal efficacies of the coated filters, filters with three different loading levels of CDots or SWCNTs (at 0.1, 0.15, or 0.2 mg) depositing on MWCNTs filter with 3 mg MWCNTs loadings were tested. Fig. 3B shows the log reductions in cell number after the filtrations using the MWCNTs–CDots filters and MWCNTs–SWCNTs coated filters with the three levels of CDots and SWCNTs loadings. The results indicated that all the filters significantly removed *E. coli* cells (*P* < 0.05) in comparison to the control samples, with the log reductions in cell number of ∼2.3 to ∼2.5 log. However, there was no statistically significant differences (*P* > 0.05) among the MWCNTs–CDots filters with different CDots loading, and among the MWCNTs–SWCNTs filters with different SWCNTs loadings, confirming the observation from the test above and proving that CDots or SWCNTs coatings did not affect MWCNTs filters' efficacies on bacterial removal.

Although MWCNTs and SWCNTs have different diameters, they have the same seamless, cylinder structure of graphene layer on the surface. SWCNTs and MWCNTs had identical zeta potential values around −4 mV when dispersing in PBS buffer solution, and around −18 mV when dispersing in cell culture medium.^41^ It suggested that the diameter of CNTs did not affect their charge distribution and the dispersion stability in PBS and cell culture medium. Besides these similarities between MWCNTs and SWCNTs, the amount of SWCNTs coating (0.1–0.2 mg) was not high enough to significantly increase the adsorption capacity and change the capability of bacterial capture by MWCNTs filters in this study, therefore, no significant difference on bacteria captures (*P* > 0.05) by MWCNTs filters and MWCNTs–SWCNTs filters was observed. Previous publications about bacterial adsorptions on SWCNTs and MWCNTs were controversial. For instance, Sweetman *et al.*^38^ reported that MWCNTs buckypapers captured more *E. coli* cells than SWCNTs buckypapers, while Choudhury *et al.* demonstrated that SWCNTs were better candidates for adsorption on microorganisms than MWCNTs.^42^ Similar controversies were also observed on biomolecules binding with CNTs at different diameters. For instance, Morikawa *et al.*^43^ observed that MWCNTs with smaller diameters had more adsorptions of osteoblast-like cells than the ones with larger diameters; on the contrary, Mu *et al.*^44^ demonstrated that MWCNTs with larger diameters (∼40 nm) generally exhibited stronger protein binding compared to those with smaller diameters (∼10 nm). These differences might be caused by different bacteria adsorption conditions, such as working on CNTs filters *vs.* in CNTs solutions, filtering cells on the CNTs filters produced with different procedures, using CNTs with different functional groups, performing in different medium solutions, *etc.*

As for CDots coatings, the average size of EDA–CDots was less than 5 nm in diameter with a carbon core of 3–4 nm in diameter in this study. CDots passivated by hydrocarbon chains on their surfaces exhibited high affinity to bacterial cells after short incubation.^45^ The bacteria–CDots binding profiles were influenced by bacterial strains, among which were involved with different lipid compositions, molecular organization, and macroscopic structure of bacterial surfaces.^45^ However, when small amount (0.1–0.2 mg) of CDots were coated on MWCNTs filters, the coating did not affect the filters' capabilities in bacteria capture.

### Effect of CDots and SWCNTs loading on bacterial inactivation

**Fig. 4:**
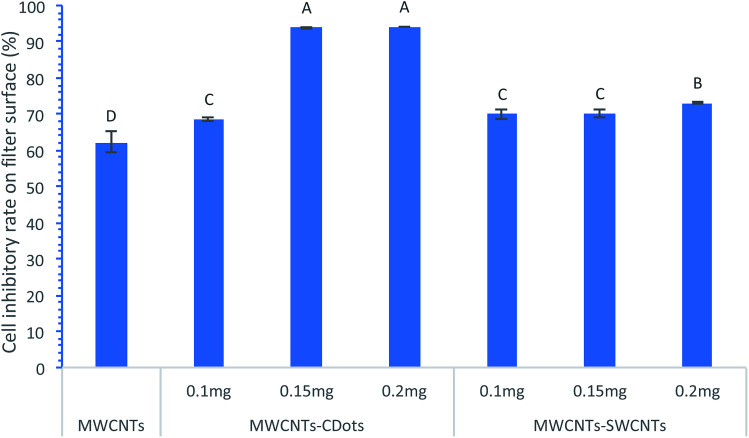
The percentages of inactivated bacterial cells on MWCNTs–CDots and MWCNTs–SWCNTs filters with different loading levels of CDots and SWCNTs, along with the MWCNTs filters for comparison. The same 3 mg MWCNTs loadings were on all the filters.

The CDots or SWCNTs coating on the MWCNTs layer was intended to afford the coated filters with antimicrobial activity which can be used to inactivate/inhibit the bacterial cells captured on the filters, as CDots and SWCNTs have both demonstrated strong antimicrobial activities against bacterial cells, and they have compatibility with MWCNTs. Fig. 4 shows the inactivation efficiencies of MWCNTs filters with three different levels of CDots and SWCNTs costing. After the cells were captured and retained on the filters for 30 min, the MWCNTs coated filters showed about 62.3% of bacteria inactivation, while the MWCNTs–CDots (0.2 mg) filters and MWCNTs–SWCNTs (0.2 mg) filters exhibited 94.2% and 73.2% inactivation efficiency, respectively, demonstrating that CDots or SWCNTs coating on the MWCNTs did afford the coated filters with antimicrobial activity. Between CDots and SWCNT coating, CDots was more effective on bacteria inactivation than SWCNTs coating, especially when both were at the same loading of 0.15 mg or 0.2 mg. Between the different levels of CDots coatings, higher CDots loading showed higher bacteria inactivation efficiency, as shown in Fig. 4, when CDots loading was increased from 0.1 mg to 0.15 mg, the bacteria inactivation percentage were significantly increased (*P* < 0.05) from 68.6% to 94.0%. Further increase of CDots coating to 0.2 mg was slightly more effective than 0.15 mg coating, but not statistically difference (*P* > 0.05). The effect of SWCNTs coating showed a slight increase trend, with the inactivation percentage only increased from 70.0% to 73.2% as SWCNTs deposit was increased from 0.1 mg to 0.2 mg. When the MWCNTs loading was 4.5 mg on all these filters, no obvious difference was observed on the inactivation efficiency in comparison to its counterpart filters with 3 mg MWCNTs loading.

The results have clearly demonstrated that the coatings of CDots or SWCNTs on the MWCNTs filters can enhance the inactivation function of the resulting filters, although studies have reported that MWCNTs filters itself without other coatings could inactivate the bacteria retained on their surfaces, despite the inactivation efficiencies being not remarkable. For example, Park reported that 83.7% of the *E. coli* cells were inactivated on a glass fiber air filter with MWCNTs deposition;^46^ Dong and Yang^4^ indicated that MWCNTs filters caused 18.9% inactivation of *B. anthracis* cells on the filter surfaces; Kang *et al.*^47^ demonstrated that MWCNTs filters led to 70% loss of metabolic activity of *E. coli* cells. The inactivation came mostly from the direct physical contact between CNTs and bacterial cells, as CNTs could damage bacterial membranes, disrupt their activities, and eventually destroy their viabilities.^47^ MWCNTs with smaller diameters generally display stronger toxicity to bacteria, while their toxicity was influenced by many factors including bacteria types and the electrostatic repulsion between MWCNTs and bacteria.^48^ SWCNTs appear to have greater antimicrobial effects than MWCNTs. The inactivation of *E. coli* cells attached to SWCNT aggregates in solution (80 ± 10%) was much higher than for the cells attached to MWCNTs (24 ± 4%).^47^ Due to the higher antimicrobial effects of SWCNTs, the additional coating of SWCNTs onto MWCNTs filters afforded higher inactivation efficiency to the cells retained on the mixed coating. In the case of CDots coating, the inactivation efficiency was enhanced by CDots as CDots have been demonstrated for very strong photo-activated antimicrobial activity.^18,49,50^ We first reported CDots' photo-activated antimicrobial activity in 2016, in which EDA–CDots with visible light illumination was observed to inactivate ∼4 log of *E. coli* cells while the same concentration of EDA–CDots without light illumination only inactivated less than 1 log of cells.^18^ A further study demonstrated the EDA–CDots' photo-activated antimicrobial activity is correlated to its optical property-fluorescence quantum yield (*Φ*_F_), with CDots with higher *Φ*_F_ having higher photo-activated antimicrobial activity.^19^ EDA–CDots' photo-activated antimicrobial activity can also be enhanced by combination with other chemicals^49^ CDots with other surface passivation, such as polyethyleneimine (PEI), also exhibited photo-activated antimicrobial activity to bacterial cells.^51^ As such, EDA–CDots was selected for coating on MWCNTs in this study. Compared to MWCNTs alone, the minimal inhibitory concentration (MIC) of CDots was 64 μg mL^−1^ on both *E. coli* and *B. subtilis* cells,^49^ while the MIC value of MWCNTs was 500 μg mL^−1^ and 2000 μg mL^−1^ when MWCNTs were dispersed in unsaturated phospholipid and in polysorbates, respectively.^52^ CDots' higher antimicrobial effects than MWCNTs, especially when the treatment was performed under light, afforded the higher bacterial inactivation efficiency on MWCNTs–CDots filters.

### Morphologies of *E. coli* cells retained on different coated filters

**Fig. 5:**
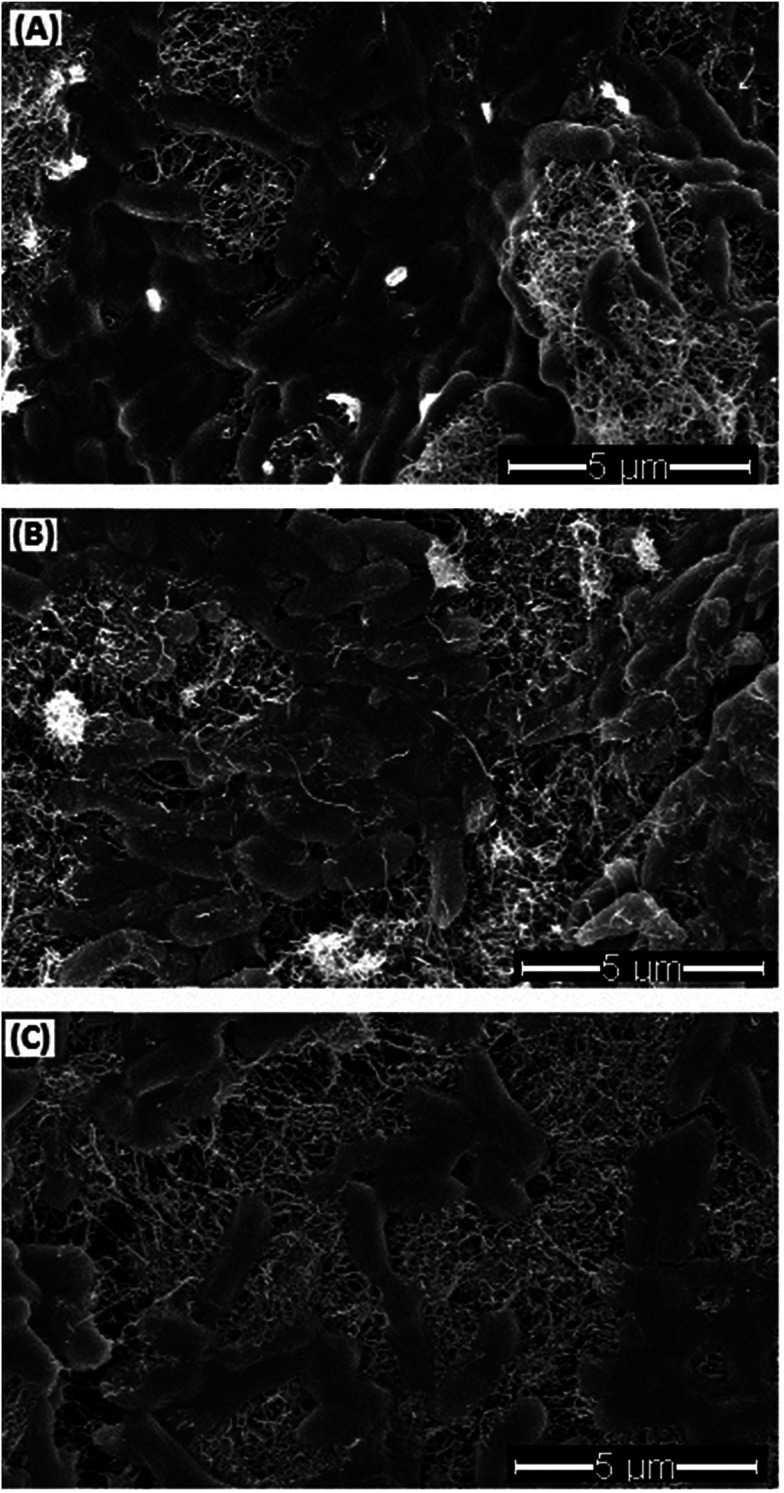
SEM images of *E. coli* cells on MWCNTs filter (A), MWCNTs–CDots filter (B), and MWCNTs–SWCNTs filter (C).

*E. coli* cells showed different morphological changes on different coated filter surfaces (Fig. 5). After 1 h retention on MWCNTs filters, the majority of the *E. coli* cells were intact and full in shape (Fig. 5A). This observation was similar to those in some of the previous studies, which demonstrated that there were no remarkable morphological changes in the cells retained on the MWCNTs filters.^4,47^ However, some other studies reported that obvious cell membrane damages were observed on bacterial cells trapped on SWCNTs or MWCNTs filters. These different observations might be due to different contact time or different filter operation procedure. Fig. 5B presents the cells on the MWCNTs–CDots filters, showing that most cells were wrapped by MWCNTs, partially flattened, and not as full as the ones on the MWCNTs filters. Dong *et al.*^49^ observed that 10 μg mL^−1^ CDots treatment on *E. coli* cells in PBS caused cells aggregation, but did not change cells' morphology. The morphological changes of cells on MWCNTs–SWCNTs filters were in between the ones on MWCNTs and MWCNTs–CDots filters (Fig. 5C). These morphological changes correlated to the cells' inactivation efficiencies by the filters, among which the MWCNTs–CDots filters showed the highest inactivation efficiency.

### Bacterial removal effect of the coated filters on Gram positive bacteria

**Fig. 6:**
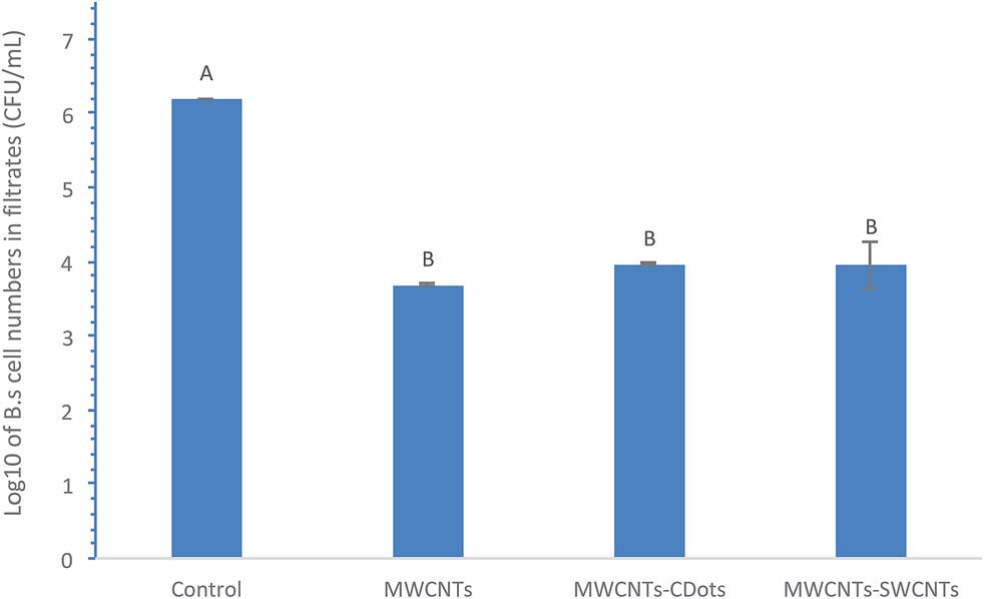
log values of *B. subtilis* cell numbers in filtrates after filtering through MWCNTs filters, MWCNT–CDots filters, and MWCNTs–SWCNTs filters.

The MWCNTs, MWCNTs–CDots, and MWCNTs–SWCNTs filters were also used to test their effects on Gram positive *Bacillus subtilis* bacteria removal. All the filters had 3 mg MWCNTs loading, while MWCNTs–CDots filters and MWCNTs–SWCNTs filters had 0.15 mg of CDots and SWCNTs coatings, respectively. Fig. 6 shows the log reduction of *B. subtilis* cells in the filtrate after the filtration with the three types of coated filters. The results indicated that all three types of filters significantly decreased *B. subtilis* cell numbers in filtrates (*P* < 0.05). *B. subtilis* cells were decreased from 6.19 log to 3.68 log by filtering through MWCNTs filters, with a reduction of 2.51 log in viable cell number. This observation was close to that in our previous publication, which indicated that MWCNTs filters with 1.5 mg of MWCNTs captured 2.44 log of *B. anthracis* cells.^4^ CDots or SWCNTs coating did not evidently affect *B. subtilis* bacteria removal efficiency, similar to the observations on *E. coli* cells. Compared to filtering *E. coli* cells, the filters showed similar efficacies in removal of *B. subtilis* cells. This is consistent with other observations that bacterial adsorption to MWCNTs occurred spontaneously in solution, and MWCNTs' adsorption capacities were nearly the same regardless of the types of strains.^53^

## Conclusions

This study demonstrated that incorporation of antimicrobial materials in MWCNTs-coated filters afforded these filters with dual functions: removal of bacterial cells from aqueous solution and inactivation of cells retained on the coated filters. Bacterial removal efficiency was largely related to the loading level of MWCNTs on the base membranes, but was not affected by the loading level of CDots or SWCNTs; whereas the inactivation function of the coated filters was related to the loading level of CDots or SWCNTs as they dominated antimicrobial activity on the filters. MWCNTs filters with 4.5 mg MWCNTs loading achieved bacterial removal from water at 5.41–6.41 log reduction on *E. coli* cells or *B. subtilis* cells, which is considered highly effective. Additional coating of 0.2 mg CDots or SWCNTs on MWCNTs filters could inactivate more than 90% and more than 70% of the cells retained on the filter, respectively, affording the filters with considerable bacterial inactivation function. The filters developed in this study have the application potential to remove both Gram positive and Gram negative bacteria cells in aqueous solution. Such multifunctional features of the coated filters are beneficial to many biological applications, including bacteria removal, isolation, concentration, water purification, detection, and decontamination of pathogens.

## Conflicts of interest

There are no conflicts of interest to declare.

## Supplementary Material
